# Delayed diagnosis and clinical course of alpha-mannosidosis: A retrospective study of 25 patients with varying severity

**DOI:** 10.1016/j.gimo.2025.103465

**Published:** 2025-10-13

**Authors:** James H. Nurse, Julia B. Hennermann, Marco A. Curiati, Nato Vashakmadze, Christina Lampe, Marina Magalhães, Sarah Donoghue, Maria Carmo Macário, Vasilica Plaiasu, Gal Tunde, Karolina M. Stepien

**Affiliations:** 1 University Hospital Southampton NHS Foundation Trust, Southampton, United Kingdom; 2 Villa Metabolica, Center for Pediatric and Adolescent Medicine, University Medical Center Mainz, Mainz, Germany; 3 Universidade Federal De São Paulo, São Paulo, Brazil; 4 Petrovsky National Research Center of Surgery, Moscow, Russia; 5 Pediatrics Department, Pirogov Russian National Research Medical University, Moscow, Russia; 6 Department of Pediatric Neurology, Muscular Diseases and Social Pediatrics, Center for Rare Diseases, University Hospital of Giessen/Marburg, Giessen, Germany; 7 Centro Hospitalar Universitário do Porto, Portugal; 8 Metabolic Diseases Unit, Royal Melbourne Hospital, Parkville, VIC, Australia; 9 Department of Neurodevelopmental Disability and Rehabilitation, Murdoch Children’s Research institute, Parkville, VIC, Australia; 10 Hospital da Universidade de Coimbra, ULS, Coimbra, Portugal; 11 Regional Centre of Medical Genetics, INSMC Alessandrescu-Rusescu, Bucharest, Romania; 12 Iuliu Hațieganu University of Medicine and Pharmacy, Cluj-Napoca, Romania; 13 Salford Care Organization, Northern Care Alliance NHS Foundation Trust, Salford, United Kingdom

**Keywords:** Alpha-mannosidosis, Delayed diagnosis, Disease severity, Enzyme replacement therapy, Lysosomal storage disorder

## Abstract

**Purpose:**

Alpha-mannosidosis is an ultrarare lysosomal storage disorder characterized by considerable diagnostic delays due to symptom heterogeneity and disease rarity. This study evaluated disease manifestation and clinical course between patients with varying disease severity to identify factors causing delayed diagnosis.

**Methods:**

Retrospective chart data were collected from 25 patients diagnosed at age ≥ 16 years categorized as having mild (*n* = 14) or moderate-severe disease (*n* = 11) across 11 centers in 7 countries.

**Results:**

Diagnostic delays were longer in patients with mild versus moderate-severe disease (mean [range]: 26.8 [10-46] vs 19.7 [14-30] years). Hearing impairment and learning/communication disability were common first signs in patients with mild and moderate-severe disease, respectively. Learning/communication disability was the most common key diagnostic sign for both groups. Symptoms appeared later in those with mild disease, although disease presentation was similar. Genetic testing confirmed diagnosis in 90% of patients; 64% of patients with mild disease had compound heterozygous variants vs 9% of those with moderate-severe disease. Patients on enzyme replacement therapy had improved or stabilized clinical conditions, indicating treatment importance regardless of age.

**Conclusion:**

This study underscores the need for improved diagnostic algorithms to facilitate timely diagnosis and treatment of patients with alpha-mannosidosis. Early suspicion and testing for alpha-mannosidosis in patients with hearing, motor, and learning impairments, along with continued reassessment of adults awaiting confirmed diagnosis are crucial.

## Introduction

Alpha-mannosidosis (OMIM 248500), an ultrarare inherited lysosomal storage disorder, affects approximately 1:1,000,000 individuals.^1^^,^^2^ It is caused by pathogenic variants in the *MAN2B1* (HGNC:6826; NM_000528.4) gene leading to deficiency of alpha-mannosidase.^2^, ^3^, ^4^ This results in the accumulation of mannose-rich oligosaccharides, thereby disrupting normal cell functions such as vesicle maturation, synaptic release, endocytosis, exocytosis, Ca^2+^ homeostasis, and autophagy.^4^^,^^5^ Alpha-mannosidosis manifests as a multisystemic disease with broad clinical symptoms.^4^, ^5^, ^6^, ^7^ These include facial dysmorphism, hearing loss, intellectual disability, developmental delay, frequent infections, ataxia, and skeletal abnormalities.^4^^,^^7^ Behavioral problems and psychotic episodes, which can occur in childhood, often become more pronounced with age.^2^^,^^8^^,^^9^ Recent research suggests that alpha-mannosidosis exists on a spectrum, and clinical use of a classification system has decreased.^7^

Alpha-mannosidosis generally manifests early; however, time to diagnosis can be delayed, ranging between 4 months and 22 years despite disease onset at ages ≤ 10 years.^10^, ^11^, ^12^ Early signs/symptoms (eg, hearing impairment), especially in milder cases, may lead to misdiagnosis owing to similarities with more common disorders (eg, mucopolysaccharidosis), ultimately increasing diagnostic delays.^2^^,^^13^

Understanding of the natural history of alpha-mannosidosis is limited,^14^ and retrospective collection of patient cases is valuable toward recognizing and describing sign and symptom progression in rare diseases, along with the development of diagnostic pathways, and treatment.^15^ Targeted therapies for alpha-mannosidosis include hematopoietic stem cell transplantation (HSCT) and enzyme replacement therapy (ERT).^4^ HSCT is generally recommended for more severe cases and early presentation of alpha-mannosidosis, with better outcomes associated with early implementation^4^; however, outcomes are variable.^2^^,^^4^ Velmanase alfa is approved by the European Medicines Agency for the treatment of nonneurological manifestations in patients with mild to moderate alpha-mannosidosis and by the US Food and Drug Administration for noncentral nervous system manifestations in adult and pediatric patients with alpha-mannosidosis.^16^^,^^17^ It has shown improved or stabilized functional outcomes and a tolerable safety profile,^18^, ^19^, ^20^, ^21^, ^22^ including in patients as young as 7 months.^22^ Early identification of alpha-mannosidosis is crucial for enabling timely therapeutic intervention.

This study reports the journey of 25 adults with delayed diagnosis of alpha-mannosidosis, making it one of the largest alpha-mannosidosis cohorts assembled without a formal registry. Here, we evaluate the differences in disease course and diagnostic pathways between patients exhibiting mild or moderate-severe disease to identify factors contributing to delayed diagnosis at age ≥ 16 years.

## Materials and Methods

This study sought case recruitment through advertisements at congresses, webinars, and symposia, inviting physicians worldwide to contribute alpha-mannosidosis cases fitting the inclusion criteria. Physicians were encouraged to invite other physicians within their networks to contribute cases; those involved in the SPARKLE registry^23^ were also invited.

Cases were included if the patient was diagnosed at ≥ 16 years of age, with either a delayed diagnosis but severe disease onset of alpha-mannosidosis in childhood, or mild disease progression with minimum/atypical symptoms in childhood. Written informed consent was obtained by the consulting physician from the patient or their legal representative, as needed according to local requirements, before sharing patient data.

Data were collected retrospectively using a data collection tool covering demographics, clinical symptoms at onset and those leading to diagnosis, molecular and biological testing (eg, oligosaccharide analyses in serum and urine, residual enzyme activity, genetic assessment), treatment status, and outcomes.

Patients were classified into 2 groups – mild alpha-mannosidosis and moderate-severe alpha-mannosidosis based on clinical profile of cases (ie, symptom manifestation/severity and age at diagnosis) and assessment by treating physician at time of project initiation.

Descriptive summaries reporting the number of patients, and mean, standard deviation, and minimum and maximum range for all continuous variables are reported.

## Results

### Patient demographics and characteristics

Retrospective chart data were collected from 25 patients who had a delayed diagnosis (ie, ≥ 16 years of age) across 11 centers in 7 countries (Australia, Brazil, Germany, Portugal, Romania, Russia, and the United Kingdom).

Of the 25 patients, 14 were identified with mild alpha-mannosidosis and 11 with moderate-severe alpha-mannosidosis (Supplemental Table 1). Most patients were female (60%, 15/25) and White (84%, 21/25; Table 1). Overall mean (range) age of disease onset was 3.9 (0-24) years, with the mild group experiencing later disease onset (6.4 [0.2-24] years) than the moderate-severe group (0.9 [0-3] years). Patients with mild disease were generally older at diagnosis (mean [range]: 33.4 [21-49] years) than those with moderate-severe disease (mean [range]: 20.6 [16-32] years). Diagnostic delay (ie, time between disease onset and diagnosis) was also longer in the mild group than in the moderate-severe group (mean [range]: 26.8 [10-46] vs 19.7 [14-30] years).

**Table 1:** Summary of demographic and medical history

Parameter	Moderate-Severe (*n* = 11)	Mild (*n* = 14)	Overall (*N* = 25)
Age at disease onset, years			
Mean (SD)	0.9 (1.0)	6.4 (8.2)^a^	3.9 (6.6)^a^
Range	0-3	0.2-24	0-24
Age at diagnosis, years			
Mean (SD)	20.6 (5.4)	33.4 (9.9)	27.8 (10.4)
Range	16-32	21-49	16-49
Diagnostic delay, years			
Mean (SD)	19.7 (5.4)	26.8 (12.1)	23.9 (10.2)
Range	14-30	10-46	10-46
Sex, *n* (%)			
Female	7 (63.6)	8 (57.1)	15 (60.0)
Male	4 (36.4)	6 (42.9)	10 (40.0)
Race, *n* (%)			
White	8 (72.7)	13 (92.9)	21 (84.0)
Asian	3 (27.3)	1 (7.1)	4 (16.0)
Consanguinity			
Yes	5 (45.5)	4 (28.6)	9 (36.0)
No	6 (54.5)	10 (71.4)	16 (64.0)
Have an affected family member			
Yes	6 (54.5)	8 (57.1)	14 (56.0)
No	5 (45.5)	6 (42.9)	11 (44.0)
Primary carer before diagnosis			
Parents/Family	3 (27.3)	3 (21.4)	6 (24.0)
Pediatric specialist^b^	2 (18.2)	4 (28.6)	6 (24.0)
Family doctor	1 (9.1)	4 (28.6)	5 (20.0)
Neurologist	3 (27.3)	2 (14.3)	5 (20.0)
Psychiatrist	0	3 (21.4)	3 (12.0)
Orthopedist	1 (9.1)	1 (7.1)	2 (8.0)
Not reported	1 (9.1)	1 (7.1)	2 (8.0)
Education			
Elementary	2 (18.2)	0	2 (8.0)
Secondary	2 (18.2)	4 (28.6)	6 (24.0)
College	0	1 (7.1)	1 (4.0)
Special needs school	5 (45.5)	5 (35.7)	10 (40.0)
Vocational	0	2 (14.3)	2 (8.0)
None^c^	2 (18.2)	2 (14.3)	4 (16.0)
Job			
Unemployed	11 (100)	6 (42.9)	17 (68.0)
Employed	0	4 (28.6)	4 (16.0)
Retired	0	1 (7.1)	1 (4.0)
Other	0	3 (21.4)	3 (12.0)
Deceased			
Yes	1 (9.1)	3 (21.4)	4 (16.0)
No	10 (90.9)	11 (78.6)	21 (84.0)

*SD*, standard deviation.

a Excludes patient ATT10 because exact age at disease onset was not available.

b Includes pediatrician, pediatric audiologist, and pedopsychiatrist.

c Includes patients who were noted as not being able to read or write.

Thirty-six percent (9/25) of patients had consanguineous parents; the proportion of patients with consanguineous parents was lower in the mild group (29%, 4/14) than the moderate-severe group (46%, 5/11). More than half of the patients (56%, 14/25) reported having an affected family member (mild, 57% [8/14]; moderate-severe, 55% [6/11]). This data set includes 5 sibling pairs (Supplemental Table 2).

Before diagnosis of alpha-mannosidosis, most patients with mild disease were under the primary care of a pediatric specialist or family doctor (each 29%, 4/14), whereas most patients with moderate-severe disease were primarily under the care of a neurologist or were not receiving specialized medical care (ie, parents/family; each 27%, 3/11; Table 1).

Across groups, up to 46% of patients attended a special needs school (mild, 36% [5/14]; moderate-severe, 46% [5/11]); the proportion of patients who attended secondary school, vocational training, or college was higher in the mild than moderate-severe group (50% [7/14] vs 18% [2/11]; Table 1).

The majority of patients with mild disease were unemployed (43%, 6/14), whereas remaining patients were employed (29%, 4/14), previously employed and retired (7%, 1/14), or involved in other work-related activities (21%, 3/14; eg, sheltered workshops or apprenticeships). This contrasted with the moderate-severe group, wherein all patients were unemployed.

### Key clinical manifestations at disease onset and diagnosis

Disease manifestations varied across patients. For this analysis, manifestations were grouped into several broad categories (Supplemental Table 3). Physicians reported the first sign of disease observed (ie, initial indicator of underlying medical condition) and key manifestations leading to diagnosis.

**Figure 1:**
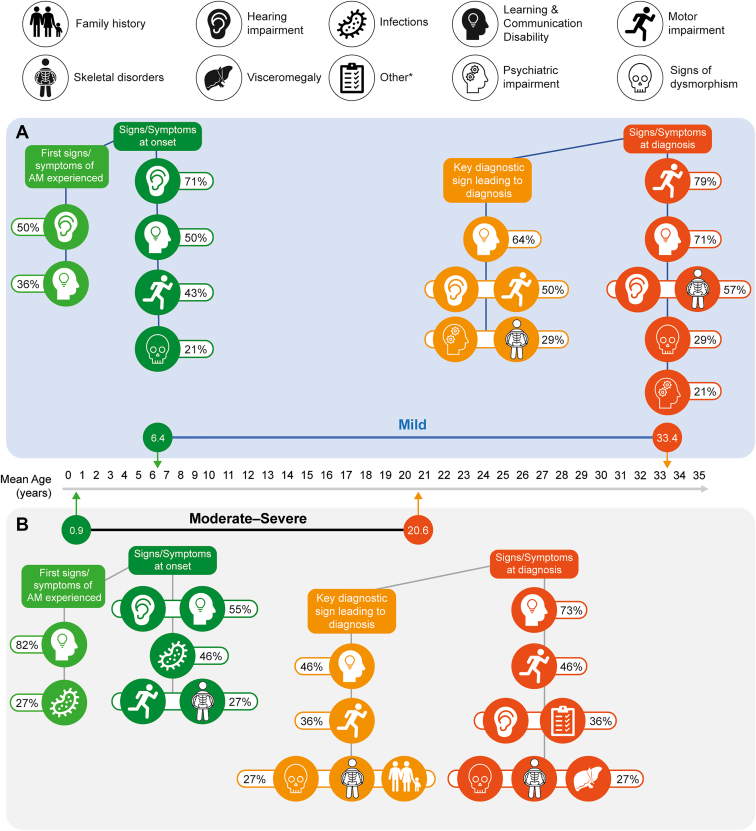
**Key disease manifestations reported by ≥20% of patients with (A) mild and (B) moderate-severe disease**. Green indicates at disease onset. Orange indicates diagnosis. ∗“Other” signs and symptoms experienced by patients with moderate-severe disease at time of diagnosis included endurance impairment and somnolence (*n* = 1), fatigue (*n* = 2), and seizures, incontinence (stool), and soft dental material (*n* = 1; dental structure described by dentist as akin to working in plasticine). *AM*, alpha-mannosidosis.

The most frequent first sign of disease observed in the moderate-severe group was learning/communication disability (82%, 9/11), followed by infections (27%, 3/11; Figure 1). This contrasted with the mild group, with half of patients exhibiting hearing impairment (50%, 7/14), followed by learning/communication disability (36%, 5/14). Additional manifestations were observed at disease onset but were not identified as the first sign/symptom (Figure 1).

In terms of key signs leading to diagnosis (Figure 1), learning/communication disability (mild, 64% [9/14]; moderate-severe, 46% [5/11]) was most frequent in both groups. In patients with mild disease, motor and hearing impairment were also frequently reported (both 50%, [7/14]), followed by skeletal and psychiatric impairments (both 29%, [4/14]). Motor impairment (36%, [4/11]), dysmorphism (27%, [3/11]), and positive family history of alpha-mannosidosis (27% [3/11]; eg, sibling receiving diagnosis) were additional key diagnosis factors for patients with moderate-severe disease. Although learning/communication disability was the most frequent key diagnostic sign for patients with mild disease, motor impairment was the most frequently reported manifestation at diagnosis in this group (79%, [11/14]).

**Figure 2:**
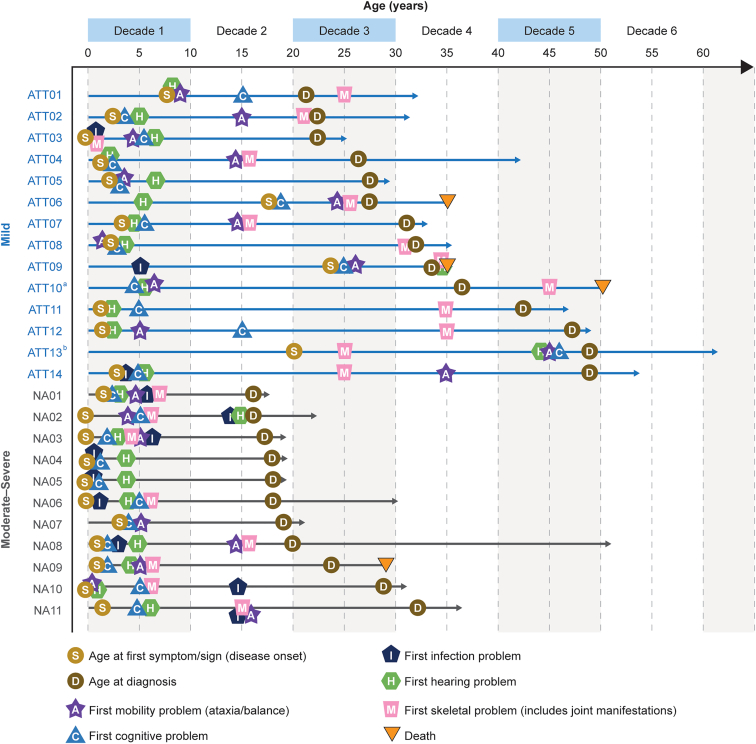
**Patient-level depiction of first symptoms**. Placement of first mobility/cognitive/infection/hearing/skeletal problem does not imply exact age experienced or order. A symptom may be reported for a given decade for a patient, but the exact age of when the symptom started may not be known. ^a^Exact age at disease onset was unknown but noted as during childhood. ^b^First symptom was not reported.

A patient-level view of the occurrence of first symptoms showed most patients first experienced multisystem symptom development within the first decade of life (Figure 2); however, patients with mild disease were more likely to experience their first cognitive, mobility (including ataxia/balance), or skeletal symptoms (including any joint manifestations) in subsequent decades. Notably, first skeletal symptoms appeared to occur closest to diagnosis in most patients with mild disease. Patients with moderate-severe disease, however, generally experienced all first symptoms (ie, hearing, cognitive, mobility, skeletal, or infection) before age 10.

Disease progression was similar between siblings, with 3/5 pairs (ATT11/ATT12; ATT05/ATT08; NA04/NA05) having identical manifestations at onset and diagnosis (Supplemental Table 2). For NA10 and NA11, 1 sibling had psychiatric manifestations and an atypical infection not seen in the other. ATT01 and NA07 were the only siblings with differing severity types. Although both exhibited ataxic gait, ATT01 presented with mild learning difficulties, earned a college degree with parental support and married. NA07, however, had more severe neurocognitive delay, attended a special needs school, and could only answer questions briefly.

Differential diagnoses were more diverse in patients with moderate-severe disease disease than mild disease (moderate-severe, 55% [6/11]; mild, 14% [2/14]; Supplemental Table 4). Diagnoses initially considered included mucopolysaccharidoses (*n* = 3), Niemann-Pick disease (*n* = 2), intellectual disability (*n* = 2), Martin-Bell syndrome (*n* = 1), Down syndrome (*n* = 1), cystic fibrosis (*n* = 1), and Scheuermann disease (*n* = 2).

Geneticists were the most important specialists leading patients to a confirmed diagnosis of alpha-mannosidosis in the moderate-severe group (64%, 7/11); in the mild group, both geneticists and neurologists were equally noted (36%, 5/11; Supplemental Table 4).

Most patients (60%, 15/25) across groups received confirmation of diagnosis (Supplemental Table 4) using a combination of 3 modalities (clinical observations, biochemical enzymatic testing, and genetic testing), followed by 24% (6/25) of patients who received confirmation through 1 modality (all instances of single confirmation were by genetic testing). Patients had confirmed diagnosis by genetic testing (96%, 24/25), enzymatic testing (72%, 18/25), and clinical observation (68%, 17/25). Of those who had their diagnosis confirmed by genetic testing, 58% (14/24) had identified the type of genetic analyses performed (single gene sequencing: 9/14; exome sequencing: 5/14). For most patients (87%, 13/15) who received confirmation by all 3 modalities, clinical observation and enzymatic testing were conducted before genetic testing.

### Clinical disease manifestation by patient age decades

Physicians were provided with a comprehensive list (Supplemental Figure 1) from which they could identify signs and symptoms that emerged and persisted or occurred intermittently across life decades.

**Figure 3:**
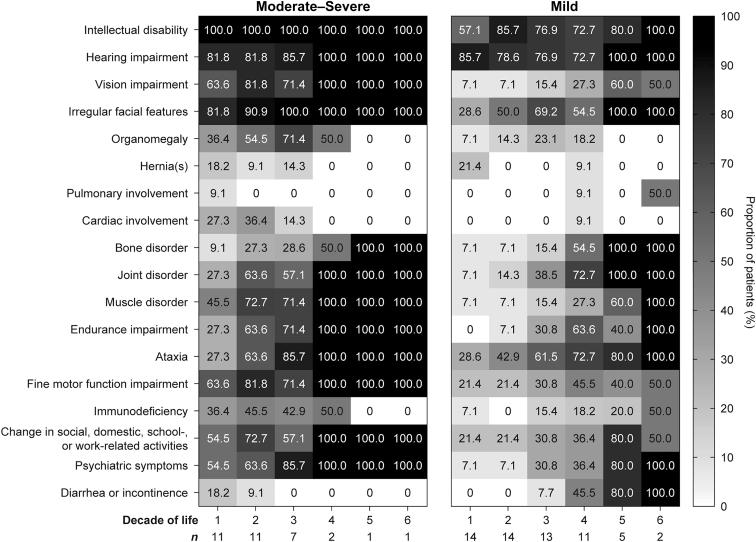
**Clinical manifestations by decade**.

Disease presentation was similar but commenced earlier in patients with moderate-severe disease than in those with mild disease (Figure 3). Intellectual disability and hearing impairment (including need for hearing aid) were apparent in over 50% of patients in both groups during the first decade of life. Irregular facial features (eg, coarse facies; Supplemental Figure 1), were highly reported in patients with moderate-severe disease within the first decade, and in those with mild disease from the second decade onward. Supplemental Figure 2 depicts facial development at different ages.

Visceromegaly, joint disorder (eg, reduced range of motion, joint pain, and joint abnormalities), muscle disorder (eg, muscle weakness, hypotonia, spasticity, contractures, and muscle pain), fine motor function impairment, and psychiatric symptoms were more common in patients with moderate-severe disease within the first 2 decades of life. The most common psychiatric symptom in patients with moderate-severe disease was behavioral problems, which was noted in over 50% of patients in the first decade and persisted across all decades. In the mild group, most psychiatric symptoms were not noted until the third decade of life, with the proportion of affected patients increasing in subsequent decades. Ataxia was present in equal proportions in both groups (mild, 29%; moderate-severe, 27%) in the first decade, increasing to over 50% during the second decade in patients with moderate-severe disease and during the third decade in those with mild disease.

### Biochemical and genetic testing

Patients with known oligosaccharide results were more likely to have had analysis conducted using urine than serum (60% [15/25] vs 16% [4/25]). Of those who had urine analyzed, most were from the mild group (73%, 11/15), and over half of these patients with mild disease had normal urine oligosaccharide results (55%, 6/11). This contrasted with the 4 patients with moderate-severe disease who had urine oligosaccharide analysis, all of whom had elevated/abnormal results.

For diagnosis confirmation, over 80% (21/25) of patients had enzymatic activity testing. Testing methods, materials (eg, plasma or leucocytes), and reference ranges varied; however, all tested patients had enzymatic activity that was well below the reported reference range except for 1 patient who had moderate-severe disease with normal alpha-mannosidase activity (dry blood spot tested). The mechanisms for this were unclear but may be related to the insufficient reliability of the assay; the patient had a confirmed genetic diagnosis. Of those with low enzymatic activity, residual enzymatic activity was below 12%, with no clear differences between groups.

**Figure 4:**
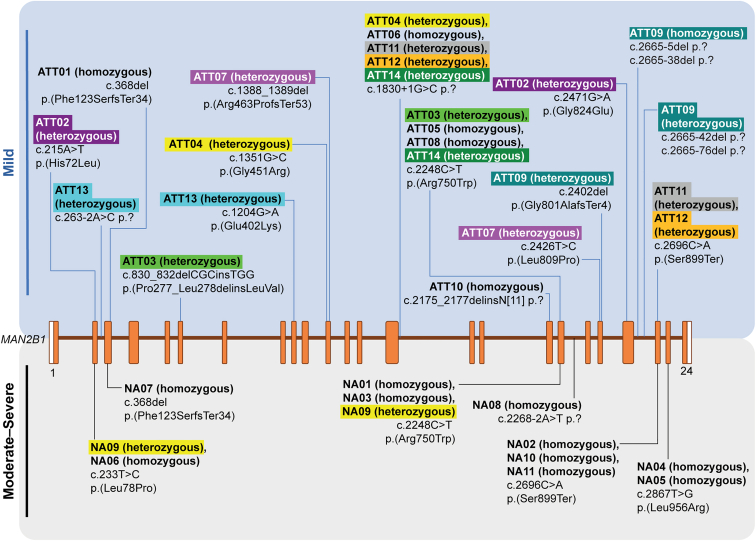
**Genetic variants**. Variants in figure are for *MAN2B1* (NM_000528.4). Orange boxes represent exons and the brown line indicates introns. Variants are labeled according to the Human Genome Variation Society (HGVS) recommendations (http://www.HGVS.org/varnomen). Each blue or black line represents a single unique variant.

Genetic analysis identified pathogenic variant clustering at the 3′ and 5′ *MAN2B1* gene ends in patients with moderate-severe disease, whereas variants in patients with mild disease were spread throughout (Figure 4). Variant classifications based on American College of Medical Genetics and Genomics/Association for Molecular Pathology guidelines^24^ are available in Supplemental Table 5. Patients with mild disease were more likely to have compound heterozygous variants (64%, 9/14), whereas most patients with moderate-severe disease were homozygous (91%, 10/11). Only 1 patient, ATT09, was noted to have 3 variants: 1 homozygous and 2 heterozygous variants. Over 50% of patients in either group had missense variants; however, patients with mild disease were also more likely to have insertion/deletion, frameshift, or splice variants (Supplemental Table 6); these variant types are mainly associated with loss of enzymatic activity.^25^

To investigate genotype-phenotype correlations between mild and moderate-severe groups, patients’ variant profiles were compared with previously described genotypic classifications: Group 1: patients with 2 null variants; group 2: patients with a genotype leading to MAN2B1 protein localization to the endoplasmic reticulum; and group 3: patients with a genotype leading to MAN2B1 localization to lysosomes.^25^ Twelve of the 25 patients (mild *n* = 9; moderate-severe, *n* = 3) could be classified. Most classified patients (8/12) were identified as group 2, with 7 patients having c.2248C>T p.(Arg750Trp) variants (either homozygous or heterozygous) and 1 with a c.2426T>C p.(Leu809Pro) variant (heterozygous). Group 2 variants were almost evenly split between patients with mild (*n* = 5) and moderate-severe (*n* = 3) disease. The remaining 4 of 9 patients with mild disease exhibited c.1830+1G>C p.?, which could be group 1 (homozygous; *n* = 1) or group 1/group 2 (heterozygous variant, dependent on allele; *n* = 3). One patient with heterozygous c.1830+1G>C p.? (allele 2) had a c.1351G>C p.(Gly451Arg) variant (allele 1) that could be classified as group 3.

### Treatment

No patients underwent HSCT. Under 40% of all patients (mild, 4/14; moderate-severe, 5/11) had received velmanase alfa treatment at any time; of those patients, 89% (mild, 4/4; moderate-severe 4/5) were still receiving velmanase alfa at data collection (Supplemental Table 7). Although age at ERT initiation was not shared, patients with mild disease receiving ERT were likely to be older at ERT initiation considering their age at diagnosis, with the mean (range) age of diagnosis of the 4 patients with mild disease receiving ERT being 27.0 (23-32) years, compared with those with moderate-severe disease (*n* = 5, 19.0 [17-24] years). Based on treating physician opinion, all patients who received velmanase alfa showed an improved or stable clinical condition. Most patients receiving velmanase alfa either had a lack of test results or availability of antidrug antibody testing.

Two patients (mild disease, *n* = 1; moderate-severe disease, *n* = 1) experienced adverse events during velmanase alfa treatment. One patient (NA03) experienced nonitching leg hyperemia that spontaneously resolved after 30 minutes during first infusion. The other patient (ATT02) had recurrent skin reactions and hypereosinophilia. Availability of antidrug antibody testing was not conducted for either patient because testing was unavailable in their respective countries.

Few patients were receiving medication for pain (mild disease, 7/14; moderate-severe disease, 1/11), prophylactic antibiotics (mild, 3/14; moderate-severe, 0/11), antipsychotics (mild, 4/14; moderate-severe, 3/11), or antidepressants (mild, 3/14; moderate-severe, 1/11; Supplemental Table 7). Up to 56% of patients (mild disease, 10/14; moderate-severe disease, 4/11) were receiving other medications/supplements (eg, iron supplements, laxatives, vitamins, and insulin).

## Discussion

This retrospective chart review provides insights into the clinical manifestations and diagnostic delays in patients with alpha-mannosidosis, an ultrarare lysosomal storage disorder. We present a cohort of 25 adults with confirmed alpha-mannosidosis across 7 countries, making it one of the largest reported alpha-mannosidosis cohorts.^25^^,^^26^ Our study evaluates the natural history of alpha-mannosidosis in adults with delayed diagnosis and compares differences in patients by disease severity.

Our findings align with previous studies, highlighting the well-known heterogeneity of alpha-mannosidosis in clinical presentation, disease progression, and genetic variation.^6^^,^^26^^,^^27^ By classifying patients into 2 groups based on disease severity (mild vs moderate-severe), we observed similar patterns of disease progression (ie, the development of signs/symptoms). However, patients with mild disease generally exhibited clinical manifestations later in life (second or third decade) than those with moderate-severe disease, who typically showed signs in the first to second decade. Patients with mild disease were also more likely than those with moderate-severe disease to exhibit psychiatric impairment, leading to diagnosis. This classification aids in understanding the timing and progression of alpha-mannosidosis, which can improve management and treatment planning.

Many patients with rare diseases face significant diagnostic delays, with some never receiving a diagnosis in their lifetime, despite approximately 70% to 75% having childhood onset.^28^, ^29^, ^30^ Patients in this study experienced delays averaging 20 to 27 years, with longer delays in the mild disease group. The retrospective nature of the study, however, makes it unclear how many physicians were consulted before diagnosis; it is likely that most patients encountered multiple specialists because of their diverse symptoms. A recent study reported that patients who saw multiple professionals were more likely to have experienced diagnostic delays.^28^ Diagnostic delays may also be exacerbated by issues relating to the health system, with a study finding an average delay of 4.3 years from first medical contact to confirmed diagnosis in patients with rare diseases.^28^ Health system factors contributing to delays may stem from inadequate physician understanding of rare diseases, availability of diagnostic resources, such as genetic testing, and prolonged waiting periods to consult specialists.^28^ Additional factors to delay included age at perceived symptom onset (children), sex (being female), and a positive family history.^28^ Undiagnosed children and newly presenting adults may lose advocates seeking treatment answers because metabolic disorders are often perceived as a childhood issue. Discussion of family life, relationships, and pregnancy are also relevant to patients but are not reported in alpha-mannosidosis. In this cohort, 3 patients with mild disease had plans for pregnancy or had been pregnant (1 patient had pregnancy terminated because of fetal anomaly, and another patient has 2 children); further differentiating mild cases from more severe cases.

Patients with moderate-severe disease had more conditions considered and excluded during differential diagnoses than those with mild disease, likely because of the heterogeneous nature of the disease, which can lead to misdiagnosis with more common conditions.^2^ Patients with mild disease often present with atypical symptoms (ie, minimal irregular facial features, mild learning disability, and ability to walk and go to school despite struggling with education) and slow progression of symptoms; these factors likely contributed to longer diagnostic delays in patients with mild disease. One patient was diagnosed through enrollment in the 100,000 Genomes Project^31^; previously, the patient had been seen and discharged by a pediatrician for mild learning difficulties in childhood and was later examined by a neurologist for ataxia in the second decade, receiving a misdiagnosis of Niemann-Pick disease. This highlights the project’s success in identifying conditions that might otherwise remain undiagnosed. Similar initiatives, such as the All of Us Research Program and the Generation Study, also strive to improve diagnosis and understanding of diseases.^32^^,^^33^ These findings support the importance of a multidisciplinary care approach in managing patients with ultrarare diseases, as recommended by a recent Delphi consensus.^34^

Learning/communication disability, followed by motor impairment, were the most frequent key factors leading to diagnosis in both groups. This differed slightly from the most common first signs at onset, with most patients exhibiting signs of learning/communication disability, hearing impairment, and infections generally within the first decade of life. These findings align with signs and symptoms of alpha-mannosidosis identified as being most prominent in those aged ≤10 years,^35^ suggesting that children with such symptoms should be promptly considered for alpha-mannosidosis with additional diagnostic testing. Furthermore, disease onset was typically before the age of 10 years, with patient with moderate-severe disease showing disease onset before 1 year, suggesting that newborn screening may be beneficial. The recent establishment of the International Consortium on Newborn Sequencing (ICoNSeq, www.iconseq.org) reflects growing global interest in the integration of genomic sequencing into newborn screening programs to identify treatable rare disorders at birth. For alpha-mannosidosis, the inclusion of *MAN2B1* in genomic panels could significantly reduce and improve diagnostic accuracy because molecular findings are generally more reliable than biochemical markers for this condition. Recent surveys indicate that up to 62% of rare disease experts support the inclusion of *MAN2B1* in genomic newborn screening programs.^36^ Early identification would not only enable prompt initiation of available treatments, such as ERT and HSCT but could also position eligible patients for future therapeutic options, including gene therapy.^37^ Although the long-term impact of early intervention—particularly on cognitive outcomes—remains to be fully understood,^4^ the potential to alter disease trajectory underscores the value of considering alpha-mannosidosis in expanded newborn screening initiatives.

Genetic testing was the most used method for confirming diagnoses, followed by biochemical enzymatic testing. However, genetic testing was often not the first method used, and the length of time between initial confirmatory methods and genetic testing was unclear. Factors such as access to genetic testing and inclusion of *MAN2B1* in genetic panels may also contribute to diagnostic delays; in the case of patient NA06, several genetic disorders were screened before finding a specialist who tested for alpha-mannosidosis. The absence of *MAN2B1* in gene panels suggests that a whole-genome approach may be more effective for diagnosing genetic disorders. Although diagnosis is possible without genetic testing,^3^ a recent Delphi consensus reported 100% recommendation for inclusion of genetic testing for a confirmatory diagnosis. Additionally, this study found that half of patients with mild disease exhibited normal oligosaccharide pattern when tested, highlighting the need for multiple screening methods to avoid missed diagnoses, especially in suspected mild cases.

Genetic analysis indicated that patients with mild disease were more likely to exhibit multiple pathogenic variants. The 3 most common *MAN2B1* variants, c.2248C>T p.(Arg750Trp), c.1830+1G>C p.?, and c.2426T>C p.(Leu809Pro),^25^^,^^27^ were identified in this cohort and accounted for 21%, 12%, and 2% of disease alleles in this study, which was comparable to reported literature.^25^^,^^27^ Most other variants reported here were uncommon. A previous study reporting variant analysis of 66 patients with mild alpha-mannosidosis identified correlations between genotype and phenotype, with those classified as group 3 exhibiting a milder form of disease compared with the other 2 groups.^25^ When this classification was applied to the patients in this cohort, most classified patients were group 2, and this was almost evenly split between patients with mild and moderate-severe disease. Only 1 patient could potentially be classified as group 3. However, not all patients could be classified based on their variants, and there was no in vitro experimentation to validate these classifications or investigate the additional variants in the rest of the cohort. Early genomic investigation (eg, genome, exome, lysosomal storage disorder genes, or *MAN2B1* sequencing) and biochemical diagnostics are recommended for patients with learning disability and hearing impairment to reduce diagnostic delays for alpha-mannosidosis.

Although a clear genotype-phenotype relationship following the groups defined by Borgwardt et al^25^ was not observed, this study reports 5 sibling pairs (ATT11/ATT12; ATT05/ATT08; ATT01/NA07; NA04/NA05; and NA10/NA11), 3 of whom had similar disease manifestations, whereas 2 pairs showed some differences in manifestation. All sibling pairs shared identical genetic variants. Differences in disease severity have previously been reported between siblings with alpha-mannosidosis, highlighting the importance of geneticists conducting detailed family genetic assessments after an alpha-mannosidosis diagnosis is made.^38^

Our study found that some patients with mild alpha-mannosidosis could achieve higher education levels and maintain employment, although often in adapted or less demanding roles due to cognitive and physical limitations. The lower disease burden presumably allowed patients with mild disease to participate more in daily activities and live more independently. In contrast, a higher proportion of patients with moderate-severe disease required special education services, and all patients were unemployed because of significant restrictions from their condition. These differences demonstrate the profound impact of alpha-mannosidosis severity on life course and socioeconomic status.

Although a recent Delphi consensus provides recommendations on early and routine monitoring for patients who received a diagnosis of alpha-mannosidosis,^34^ there are no clinical treatment guidelines. Therapeutic goals differ between children and adults, focusing on early treatment and developmental support in children,^4^ and preventing disease progression and improving quality of life in adults. Treatment goals for patients with mild disease are unclear but highlight the balance between disease and treatment burden in older patients. Velmanase alfa treatment has improved patient functional capacity and quality of life,^20^^,^^21^^,^^39^, ^40^, ^41^ especially in pediatric patients.^41^ Less than half of this cohort had received ERT, with those treated likely starting in their second or third decade of life. The reasons for patients not receiving ERT possibly include costs, availability, or patient preference; however, these reasons were not captured in this study and future work should aim to assess these factors. Despite delays in diagnosis and starting ERT as adults, patients showed improved or stable clinical conditions. Although it is unclear how patients improved with treatment because of the limitations of the collection tool, these outcomes still highlight treatment importance at any age. These findings are consistent with those from a recent international caregiver and patient survey, which found that untreated patients had greater deterioration across functional and quality-of-life measures than those who received HSCT or ERT, including adults on ERT who had a median age at treatment initiation of 21.5 years.^42^ This decline may also significantly affect aging parents and caregivers, although detailed exploration of this burden remains limited.^6^^,^^43^

Although adult patients benefit from treatment after late diagnosis, early diagnosis and treatment initiation are crucial to prevent disease progression. Proposals for diagnostic algorithms to facilitate timely diagnosis and early treatment initiation for patients ≤ 10 and >10 years of age was published in 2019,^35^ but impact on reducing diagnosis delays remains to be seen. A recent review by Santoro et al^2^ discusses practical approaches to reduce diagnostic delays, including the need for validated diagnostic algorithms, screening tools, and education of health care providers. Our findings also emphasize the need for robust diagnostic algorithms to facilitate early diagnosis and increase the likelihood of identifying patients with milder forms of alpha-mannosidosis.

Although this study represents one of the largest reported adult cohorts with alpha-mannosidosis and aims to highlight differences in disease course and diagnostic pathways, there are several limitations to consider when interpreting its findings. The study’s retrospective chart review design relies on data that are prone to inconsistencies and biases, including recall and selection bias. This reliance on preexisting medical records can lead to inaccurate or incomplete information, potentially skewing the results. The use of free-text data gathering further introduces variability in reporting signs and symptoms, which can affect the consistency and reliability of the reported symptoms and may complicate the comparison of manifestations across patients. Although we have attempted to standardize the data extraction process, some variability remains. Grouping patients as having mild or moderate-severe disease was based on authors’ assessment of clinical profiles. This subjective assessment may not capture the full spectrum of disease severity and could introduce bias. Testing values and reference ranges were not standardized across the cohort, complicating the comparison of enzymatic activity between groups. Although treatment outcome measures in this study were not standardized and were based on individual physician assessments of clinical stability or improvement, these observations nonetheless provide valuable real-world insights into patient responses to therapy. Similarly, although reasons for initiating or not initiating ERT were not systematically captured, it is likely that factors such as treatment availability, cost, and patient/caregiver preference played a role. Recognizing these limitations, future research should incorporate standardized outcome measures and structured data collection tools to enhance comparability across studies and further explore the contextual factors that influence treatment decisions. Despite these challenges, these findings offer important perspectives on clinical practice and patient experiences in the management of alpha-mannosidosis.

This study provides important insights into the clinical presentation and diagnostic delays associated with alpha-mannosidosis in 25 adult patients and reports findings based on disease severity. These results contribute to the existing body of knowledge regarding the natural history of alpha-mannosidosis and highlight the importance of patient case collection regarding development of diagnostic pathways and treatments in rare diseases. Hearing impairment, learning/communication disability, and motor impairment, particularly in children, should be regarded as useful flags to raise early clinical suspicion of alpha-mannosidosis and improve the chances of preserving a patient’s quality of life and life expectancy.

## Data Availability

The original contributions presented in the study are included in the article and Supplemental materials. Further inquiries can be directed to the corresponding author.

## Conflict of Interest

Julia B. Hennermann received consulting fees/speaker honoraria and/or travel expenses from 10.13039/100007560Chiesi Farmaceutici S.p.A., 10.13039/100015362Amicus, 10.13039/100004329Genzyme 10.13039/100004339Sanofi, and Takeda. Marco A. Curiati reports participating in speakers’ bureau and receiving honoraria for non-CME from Sanofi Genzyme, Takeda, Biomarin, Chiesi, and Ultragenyx. Nato Vashakmadze has received travel grants and speaker honoraria from 10.13039/100004339Sanofi, Takeda, Biomarin, 10.13039/100019719Chiesi, and 10.13039/100004325AstraZeneca. Christina Lampe has received travel grants, speaker's fees, and honorarium for advisory boards from Biomarin, 10.13039/100019719Chiesi, 10.13039/100015362Amicus, 10.13039/100006396Alexion, 10.13039/100004339Sanofi, Takeda, and 10.13039/501100004095Kyowa Kirin. Vasilica Plaiasu has received travel grants, speaker’s fees and honorarium for advisory boards from 10.13039/100019719Chiesi, Takeda (10.13039/100007343Shire), 10.13039/100008484BioMarin, and 10.13039/100004339Sanofi. Karolina M. Stepien is a member of advisory board and received consulting fees, research funding, and/or travel reimbursement from 10.13039/100008484BioMarin, 10.13039/100007560Chiesi Farmaceutici S.p.A, 10.13039/100013995Sanofi Genzyme, and Takeda. All other authors declare no conflicts of interest.
