# Supplementary material for: Delayed diagnosis and clinical course of alpha-mannosidosis: A retrospective study of 25 patients with varying severity
Source: Genet Med Open. 2025 Oct 13;3:103465. doi: 10.1016/j.gimo.2025.103465 (PMC12666804; doi:10.1016/j.gimo.2025.103465)
Supplement: Supplemental Figures [file mmc1.pdf]

Delayed diagnosis and clinical course of alpha-mannosidosis: A retrospective study of 25 patients with varying severity

Keywords

alpha-mannosidosis, lysosomal storage disorder, delayed diagnosis, disease severity, rare disease, enzyme replacement therapy.

The following Supplemental Tables are in the accompanying excel sheet.

Supplemental Table 1. *Demographic and medical history by patient*
Supplemental Table 2: *Clinical manifestations in siblings*
Supplemental Table 3: *Categorization of verbatim terms*
Supplemental Table 4: *Diagnostic pathway*
Supplemental Table 5: *MAN2B1 variants*
Supplemental Table 6: *Genetic variant type*
Supplemental Table 7: *Treatment*

A list of references cited in the Supplemental Tables are located in the Information tab of the excel sheet, and at the end of this document.

Supplemental Figures

***Supplemental Figure 1**: Comprehensive list of clinical manifestations by decade*

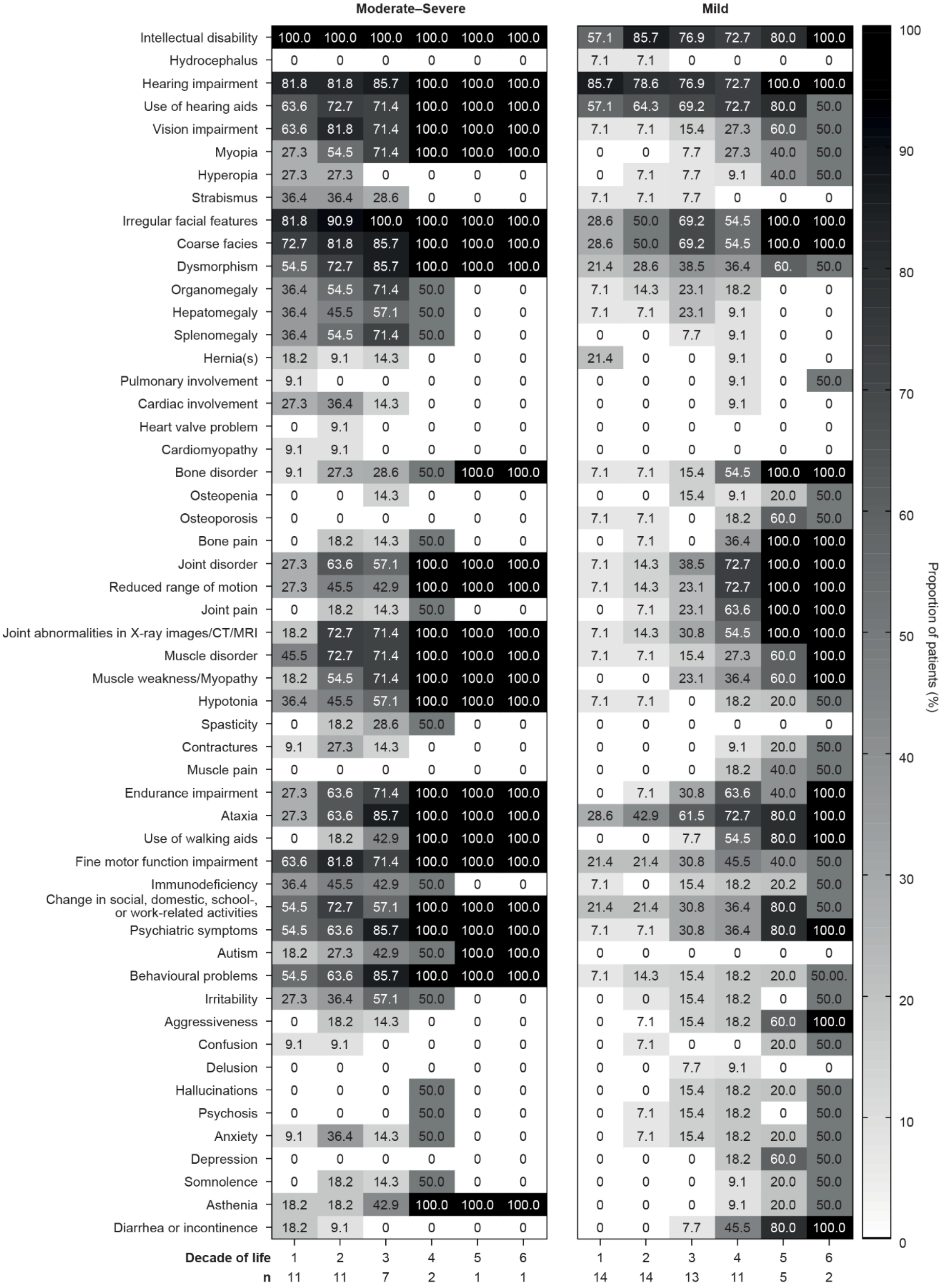

Supplemental Figure 2: *Patient facial development at different ages*

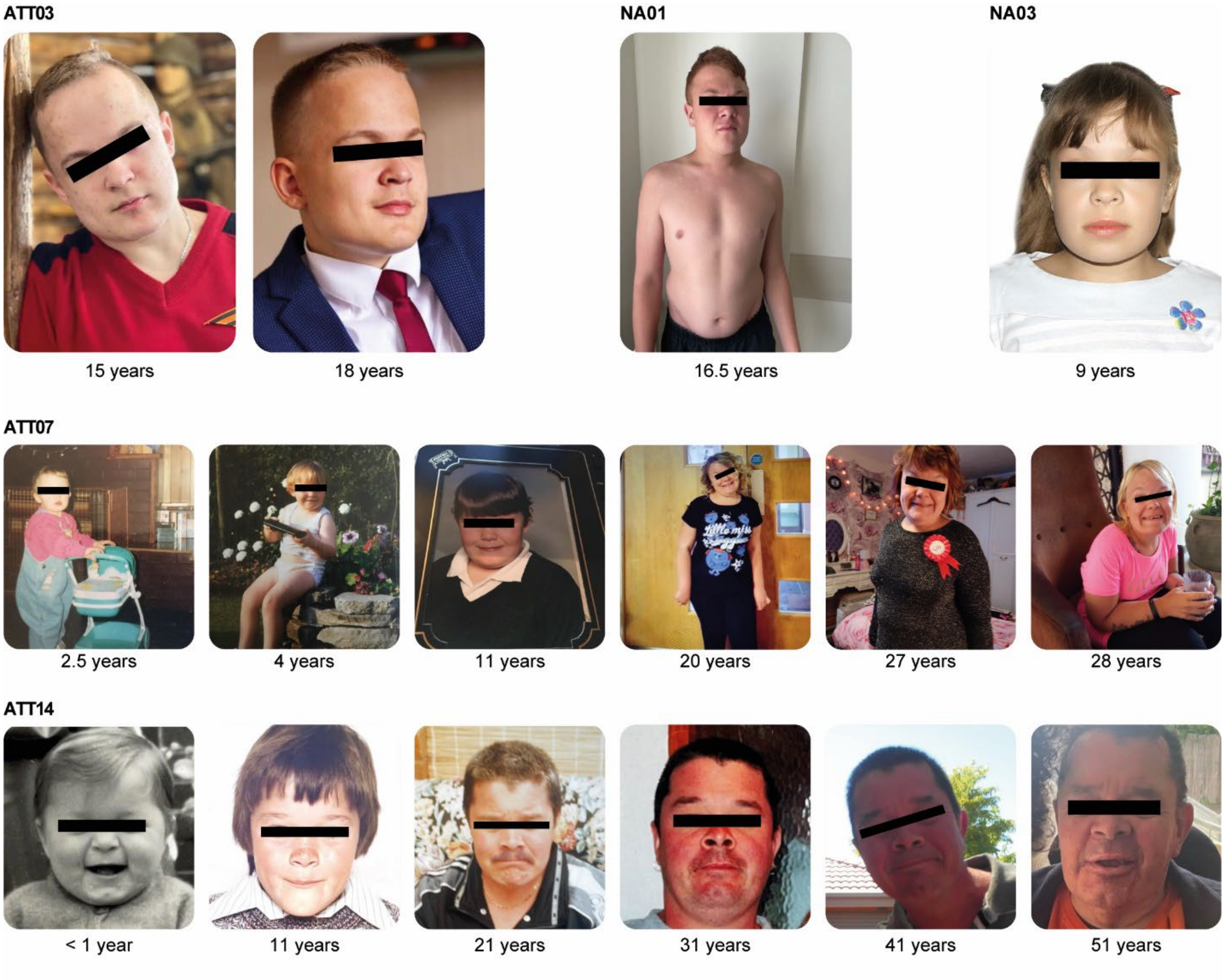

References (for Supplemental Tables)

1. Richards S, Aziz N, Bale S, et al. Standards and guidelines for the interpretation of sequence variants: a joint consensus recommendation of the American College of Medical Genetics and Genomics and the Association for Molecular Pathology. Genet Med. 2015;17(5):405-424.

2. Borgwardt L, Stensland HM, Olsen KJ, et al. Alpha-mannosidosis: correlation between phenotype, genotype and mutant MAN2B1 subcellular localisation. Orphanet J Rare Dis. 2015;10:70.

3. National Center for Biotechnology Information. ClinVar; [VCV000001687.74], https://www.ncbi.nlm.nih.gov/clinvar/variation/VCV000001687.74 (accessed July 18, 2025).

4. National Center for Biotechnology Information. ClinVar; [VCV000370174.14], https://www.ncbi.nlm.nih.gov/clinvar/variation/VCV000370174.14 (accessed July 18, 2025).

5. National Center for Biotechnology Information. ClinVar; [VCV000208280.11], https://www.ncbi.nlm.nih.gov/clinvar/variation/VCV000208280.11 (accessed July 18, 2025).

6. National Center for Biotechnology Information. ClinVar; [VCV000892434.6], https://www.ncbi.nlm.nih.gov/clinvar/variation/VCV000892434.6 (accessed July 18, 2025).

7. Varsome. Variant; [chr19-12665420-A], https://varsome.com/variant/hg38/chr19-12665419-GA-G? (accessed July 18, 2025).

8. National Center for Biotechnology Information. ClinVar; [VCV001066687.6], https://www.ncbi.nlm.nih.gov/clinvar/variation/VCV001066687.6 (accessed July 18, 2025).

9. National Center for Biotechnology Information. ClinVar; [VCV000001684.10], https://www.ncbi.nlm.nih.gov/clinvar/variation/VCV000001684.10 (accessed July 18, 2025).

10. Varsome. Variant; [chr19-12648368-C-T], https://varsome.com/variant/hg38/chr19-12648368-C-T? (accessed July 18, 2025).

11. Varsome. Variant; [chr19-12663394-GCG-CCA], https://varsome.com/variant/hg38/chr19-12663394-GCG-CCA (accessed July 18, 2025).

12. Varsome. Variant; [chr19-12657514-C-G], https://varsome.com/variant/hg38/chr19-12657514-C-G? (accessed July 18, 2025)

13. National Center for Biotechnology Information. ClinVar; [VCV000021207.40], https://www.ncbi.nlm.nih.gov/clinvar/variation/VCV000021207.40 (accessed July 18, 2025).

14. National Center for Biotechnology Information. ClinVar; [VCV000848454.22], https://www.ncbi.nlm.nih.gov/clinvar/variation/VCV000848454.22 (accessed July 18, 2025).

15. National Center for Biotechnology Information. ClinVar; [VCV000021210.26], https://www.ncbi.nlm.nih.gov/clinvar/variation/VCV000021210.26 (accessed July 18, 2025).

16. Varsome. Variant; [chr19-12649170-C], https://varsome.com/variant/hg38/chr19-12649169-GC-G? (accessed July 18, 2025).

17. Varsome. Variant; [chr19-12647640-C], https://varsome.com/variant/hg38/chr19-12647639-GC-G? (accessed July 18, 2025).

18. Varsome. Variant; [chr19-12647674-G-], https://varsome.com/variant/hg38/chr19-12647673-AG-A? (accessed July 18, 2025).

19. Varsome. Variant; [chr19-12647603-G], https://varsome.com/variant/hg38/NM_000528.4%3Ac.2665-5del? (accessed July 18, 2025).

20. Varsome. Variant; [chr19-12647636-G], https://varsome.com/variant/hg38/chr19-12647635-CG-C? (accessed July 18, 2025).

21. Hennermann JB, Raebel EM, Dona F, et al. Mortality in patients with alpha-mannosidosis: a review of patients' data and the literature. Orphanet J Rare Dis. 2022;17(1):287.

22. National Center for Biotechnology Information. ClinVar; [VCV002910452.2], https://www.ncbi.nlm.nih.gov/clinvar/variation/VCV002910452.2 (accessed July 18, 2025).

23. National Center for Biotechnology Information. ClinVar; [VCV000208283.10], https://www.ncbi.nlm.nih.gov/clinvar/variation/VCV000208283.10 (accessed July 18, 2025).
